# Inflammatory Fibroid Polyp of the Intrahepatic Biliary Tract: First Documented Case and Diagnostic Relevance of SpyGlass DS Cholangioscopy

**DOI:** 10.1055/a-2826-6215

**Published:** 2026-08-19

**Authors:** Meral Saraldi, Judith Knievel, Ulrich Huegle, Christoph G. Dietrich, Michael Brockmann, Arno Josef Dormann, Claus Ferdinand Eisenberger

**Affiliations:** 1 Klinik für Gastroenterologie, Gastrointestinale Onkologie, Gastrointestinale Infektionen und ErnährungsmedizinKrankenhaus Holweide 15055 Kliniken der Stadt Köln gGmbHCologneGermany; 2 Klinik für Viszeral-, Tumor-, Transplantations- und Gefäßchirurgie 15055 Kliniken der Stadt Köln gGmbHCologneGermany; 3 MVZ Wiesbaden/Rheingau-TaunusWiesbadenGermany; 4 Institut für Pathologie 15055 Kliniken der Stadt Köln gGmbHCologneGermany

**Keywords:** inflammatory fibroid polyp (IFP), SpyGlass DS Cholangioscopy, mesenchymal tumor, intrahepatic biliary mass, vanek tumor, inflammatorisch-fibroider Polyp (IFP), SpyGlass DS Cholangioskopie, mesenchymaler Tumor, intrahepatische biliäre Raumforderung, Vanek-Tumor

## Abstract

A 19-year-old female presented with recurrent upper abdominal pain, pruritus, and jaundice. Imaging and endoscopic evaluation revealed a high-grade biliary stricture in the hilum and left hepatic bile duct with a tumor-like lesion. Repeated brush and fluoroscopic guided biopsies failed to yield a definitive diagnosis. Using SpyGlass DS single-operator cholangioscopy, targeted biopsies were obtained, indicating a benign mesenchymal neoplasm. Due to persistent cholestasis and suspected malignancy, left hemihepatectomy was performed. Final histopathology revealed an inflammatory fibroid polyp of the intrahepatic bile duct – a previously undocumented localization. The patient recovered fully after surgery. This case highlights that inflammatory fibroid polyps can present as biliary tumors and underscores the diagnostic value of SpyGlass DS cholangioscopy in evaluating indeterminate biliary strictures.

## Zusammenfassung

Eine 19-jährige Patientin stellte sich mit rezidivierenden Oberbauchschmerzen, Juckreiz und Ikterus vor. Bildgebung und Endoskopie zeigten eine hochgradige Gallengangsstenose im Hilus und linkshepatisch mit tumorartiger Raumforderung im Hilusbereich. Bürstenzytologie und fluoroskopisch-geführte Biopsien führten zu keiner histologischen Diagnose. Erst durch eine single-operator-Cholangioskopie mit dem SpyGlass-DS-System konnten gezielte Biopsien entnommen werden, die auf eine mesenchymale, nicht-maligne Läsion hinwiesen. Aufgrund der persistierenden Cholestase und des malignitätsverdächtigen Befundes erfolgte eine Hemihepatektomie links. Die endgültige Diagnose ergab einen inflammatorisch-fibroiden Polypen (inflammatory fibroid polyp) der intrahepatischen Gallenwege – ein bislang in der Literatur in dieser Lokalisation nicht beschriebener Befund. Die Patientin erholte sich vollständig nach der Operation. Dieser Fall zeigt, dass inflammatorisch-fibroide Polypen, die vorwiegend im Gastrointestinaltrakt lokalisiert sind, auch in den intrahepatischen Gallenwegen auftreten können und die modulare single-operator-Cholangioskopie eine entscheidende Rolle bei der Diagnose unklarer biliärer Stenosen spielt.

## Introduction

Inflammatory fibroid polyps (IFPs), also known as Vanek's tumor, are rare, benign mesenchymal lesions of the gastrointestinal tract, most commonly found in the gastric antrum and ileum 1 2 3 . They typically present as solitary, submucosal, polypoid masses composed of spindle-shaped stromal cells, a fibromyxoid stroma, and a prominent inflammatory infiltrate rich in eosinophils 3 4 5 6 . The classic histologic feature is perivascular “onion-skinning” of spindle cells, although this is not universally present 3 7 .

The etiology of IFPs remains unclear, but molecular studies have identified activating mutations in the platelet-derived growth factor receptor alpha (PDGFRA) gene in a significant proportion of cases, supporting a neoplastic rather than purely reactive origin 7 8 9 . These mutations are similar to those seen in gastrointestinal stromal tumors (GISTs), but IFPs lack c-KIT mutations and do not behave aggressively 4 7 9 .

Clinically, IFPs may be asymptomatic or present with nonspecific symptoms such as abdominal pain, gastrointestinal bleeding, or, in the small intestine, intussusception and acute abdomen 1 2 3 . Diagnosis is established by endoscopic or surgical biopsy and histopathological examination. Immunohistochemistry typically shows positivity for CD34 and variable expression of actin, with negativity for c-KIT and S100 3 4 6 .

Treatment is usually by endoscopic resection, which is curative; surgical intervention is reserved for cases with complications such as intussusception or obstruction 1 2 10 . Recurrence is exceedingly rare, and malignant transformation has not been reported 2 3 10 .

## Case Presentation

A 19-year-old female patient complained about recurrent upper abdominal pain, itching and jaundice since several months until her first gastroenterology consultation in early 2020. She presented with elevated levels of serum bilirubin, alanine aminotransferase (ALT), aspartate aminotransferase (AST), gamma glutamyl transferase (GGT), and alkaline phosphatase (ALP) ( Table 1 ). The abdominal ultrasonography revealed an intra- and extrahepatic cholestasis, confirmed by an endosonography and magnetic resonance imaging (MRI/MRCP) ( Fig. 1 ). The cholestasis was predominantly in the left liver lobe with evidence of a tumor involving the hilum and left hepatic bile duct. An initial ERC confirmed the mass in the hilum with left hepatic cholestasis ( Fig. 2 ). Brush biopsies were obtained, the stenosis was dilated, and a plastic stent (7 Fr/14 cm) was placed in the left hepatic bile duct. The cytology did not reveal malignancy, but a definitive diagnosis was not established either. Due to an acute pain attack and persistent cholestasis, ERC was repeated after 10 days. The stent was removed, forceps biopsies were taken under fluoroscopic control, and a new stent was implanted. Once again, no histologic diagnosis could be obtained from the collected specimens.

**Table:** **Table 1** Laboratory findings.

Laboratory findings			
**Initial presentation**			
	Result	Units	Ref. interval
**Total bilirubin**	**6.76**	mg/dl	< 1.1
**AST**	**149**	U/l	< 35
**ALT**	**195**	U/l	< 35
**GGT**	**580**	U/l	< 40
**Two months after hemihepatectomy**			
	Result	Units	Ref. interval
**Total bilirubin**	0.27	mg/dl	< 1.2
**AST**	33	U/l	< 35
**ALT**	31	U/l	< 40
**GGT**	49	U/l	< 23
AST: aspartate aminotransferase; ALT: alanine aminotransferase; GGT: gamma glutamyl transferase			

**Fig. 1:**
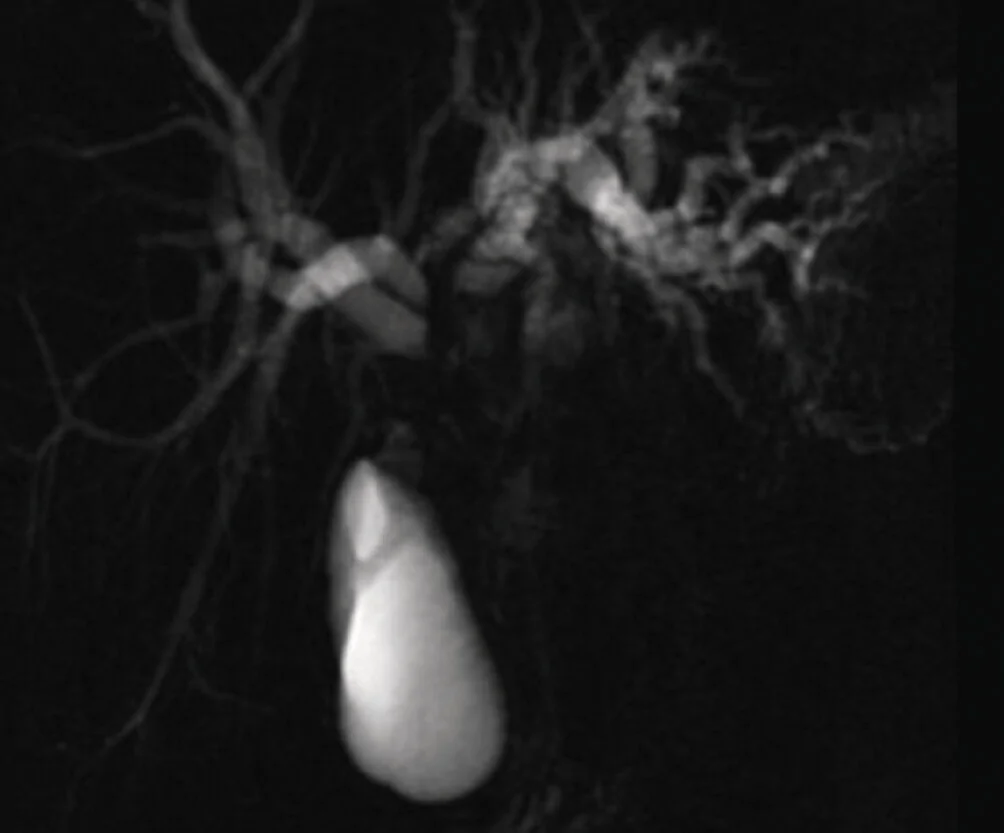
MRI/MRCP. Severe left hepatic cholestasis. Source: Bethlehem Gesundheitszentrum Stolberg GmbH, Stolberg.

**Fig. 2:**
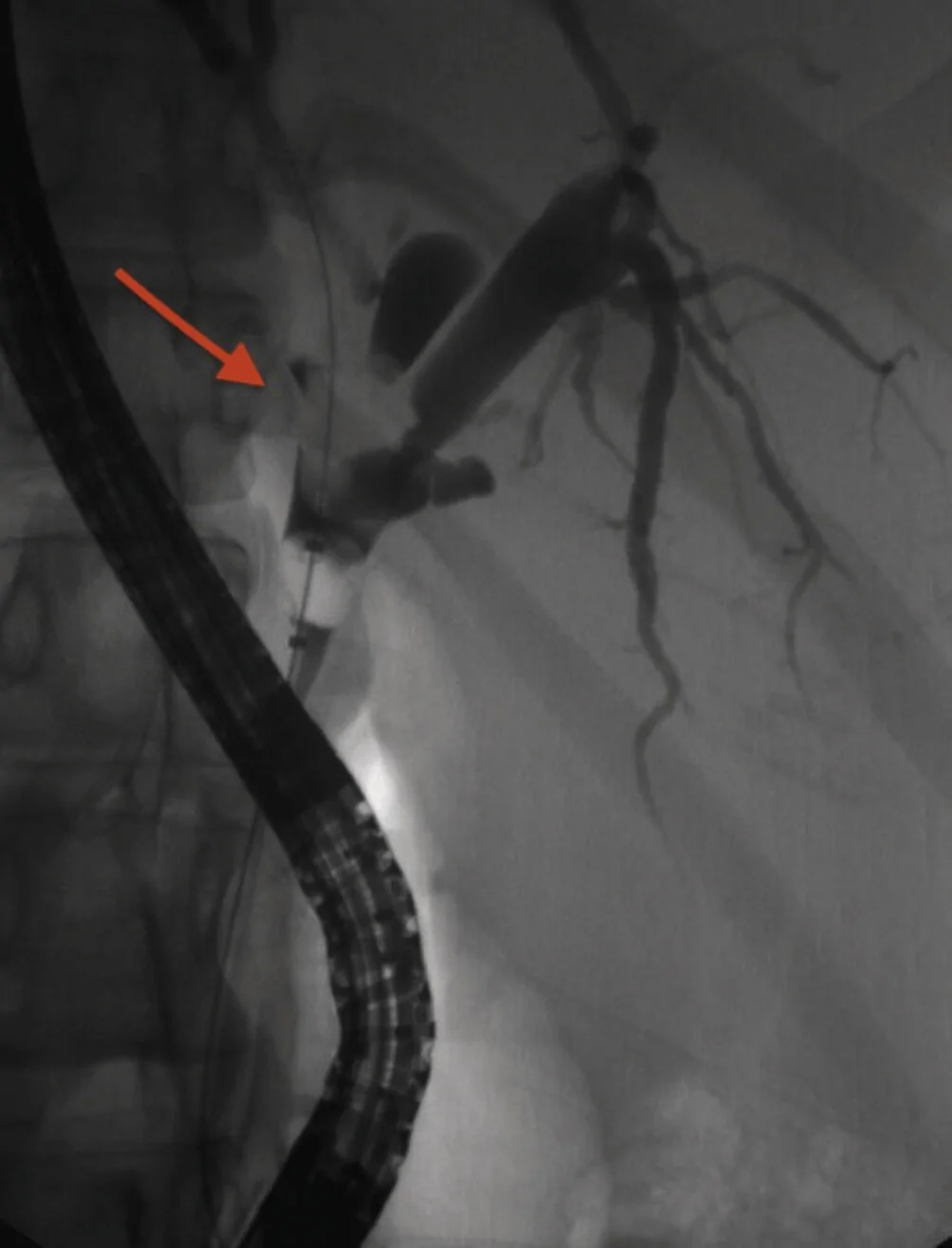
ERC, balloon occlusion cholangiography. Hilar mass, extending in the left hepatic bile system.

The patient was referred to our tertiary care center for further evaluation of the suspicious biliary stenosis. After confirming the previous findings with ongoing cholestasis, we performed an ERC with SpyGlass DS single-operator cholangioscopy. Fluoroscopy revealed an obstructing hilar tumor extending from 2 cm below the hilum up to 4 cm into the left hepatic bile duct, causing a high-grade stenosis ( Fig. 3 and Fig. 4 ). In the direct visualization we saw a bulky tumor with prominent vascularization ( Fig. 5 ), not suspicious for malignancy. Several biopsies were taken with the SpyBite biopsy forceps followed by implantation of a long plastic stent into the left bile duct. Histopathology and immunohistochemistry revealed a mesenchymal neoplasia with no evidence of proliferation or malignancy ( Fig. 6 , Fig. 7 , Fig. 8 ).

**Fig. 3:**
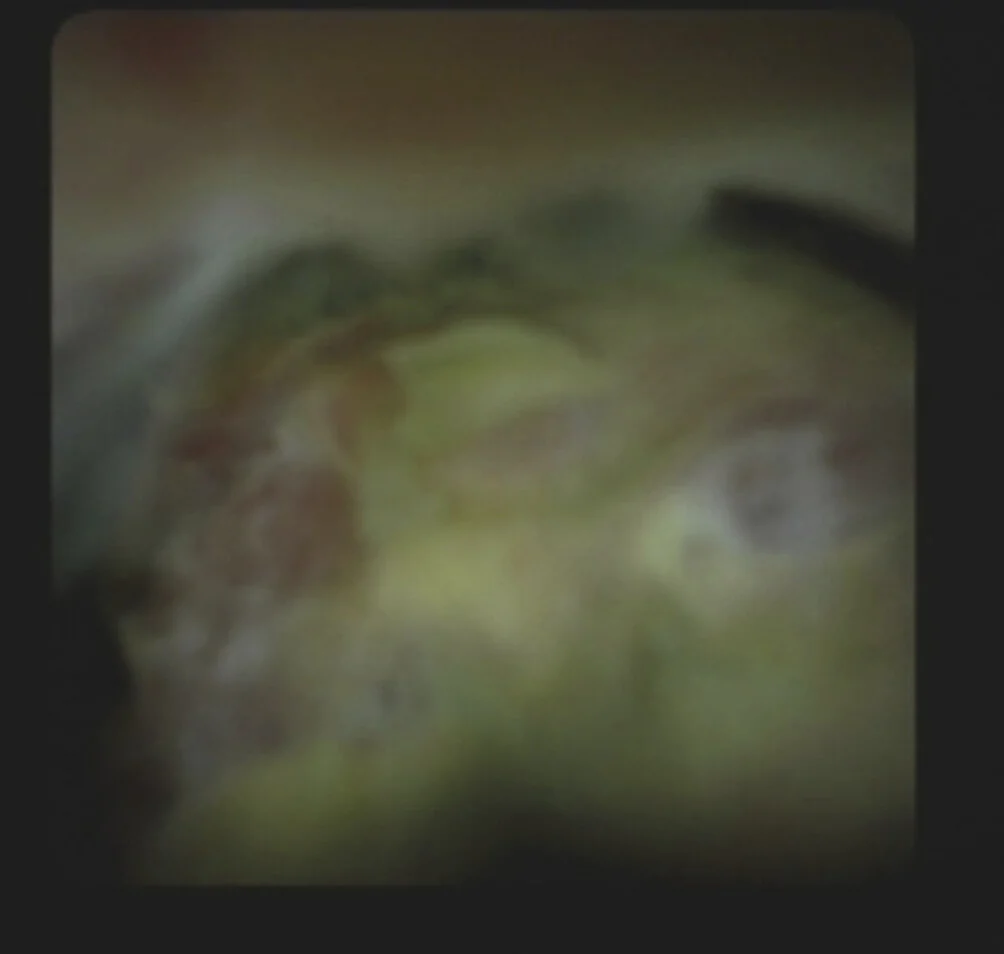
Single Operator Cholangioscopy. Bulky and obstructing inflammatory fibroid polyp in the hilum / left hepatic bile duct.

**Fig. 4:**
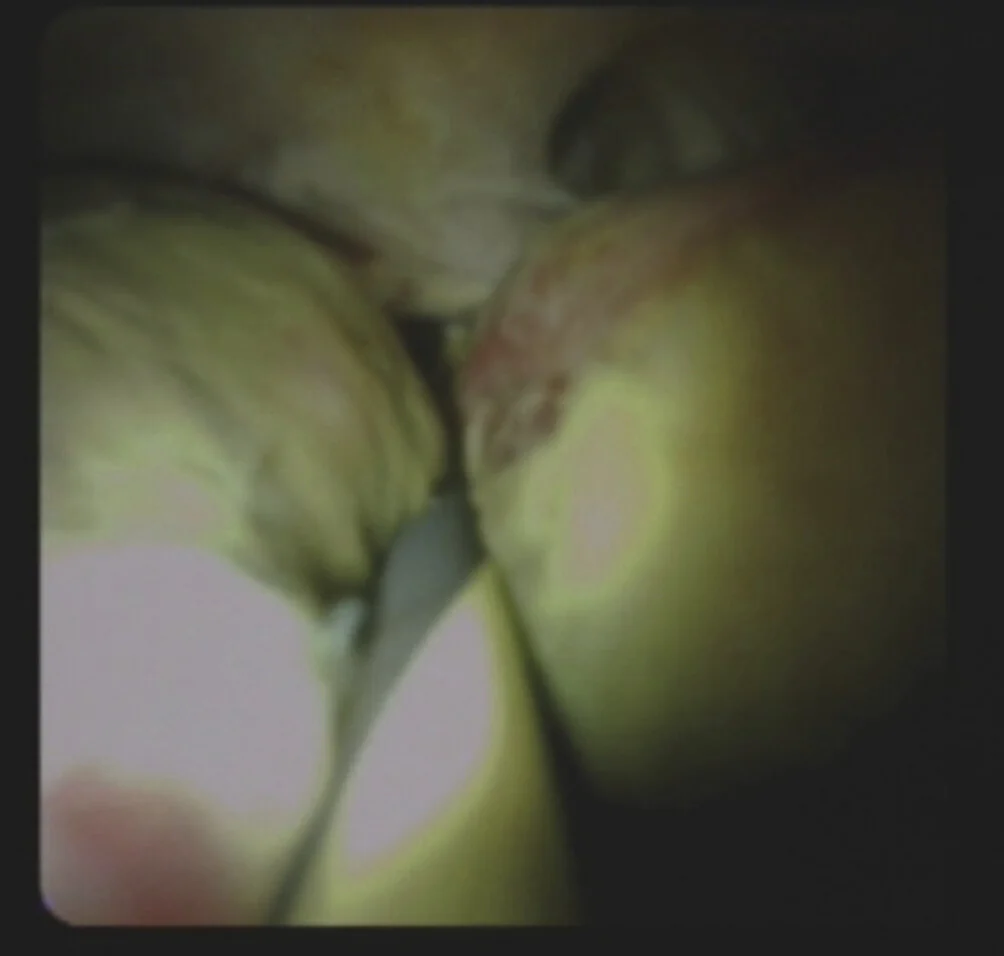
Single Operator Cholangioscopy. Bulky and obstructing inflammatory fibroid polyp in the hilum / left hepatic bile duct.

**Fig. 5:**
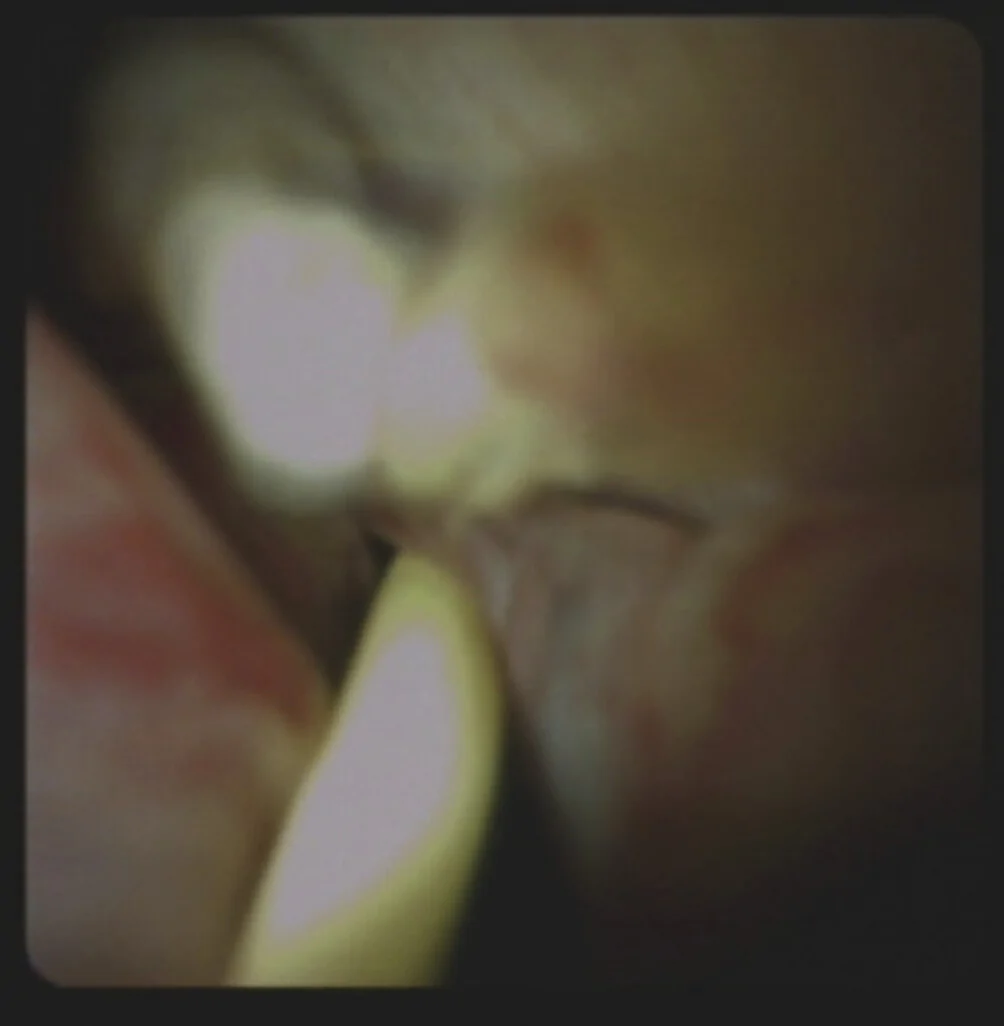
SOC. Neovascularisation of the inflammatory fibroid polyp in the hilum / left hepatic bile duct.

**Fig. 6:**
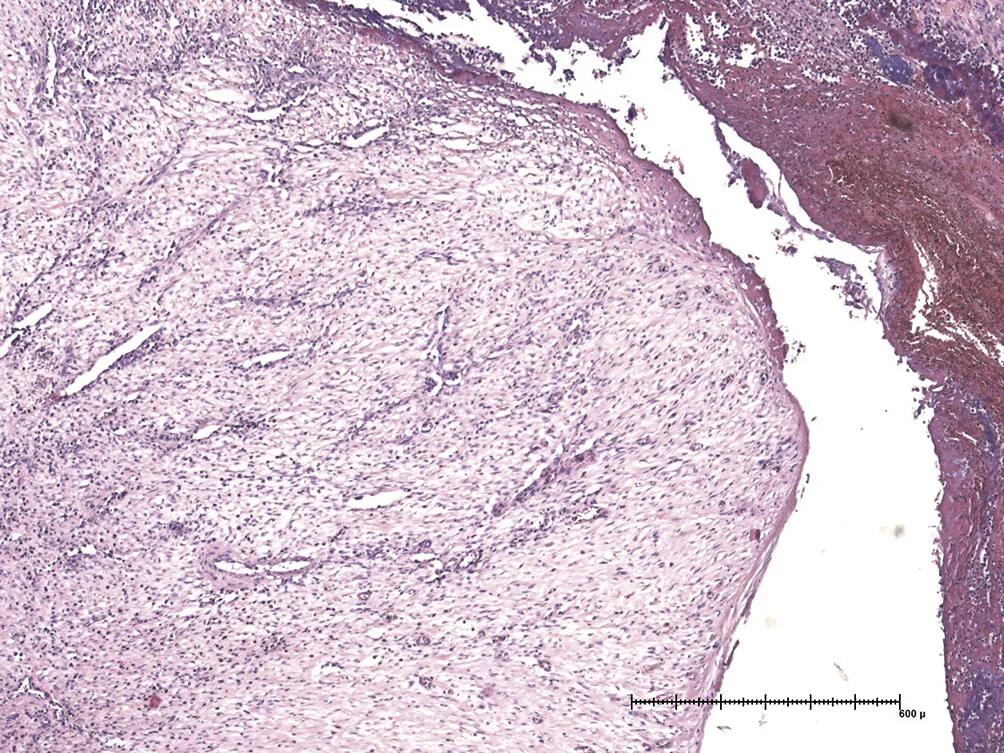
Inflammatory Fibroid Polyp. Branch of the lesion in the lumen of the dilated left hepatic duct.

**Fig. 7:**
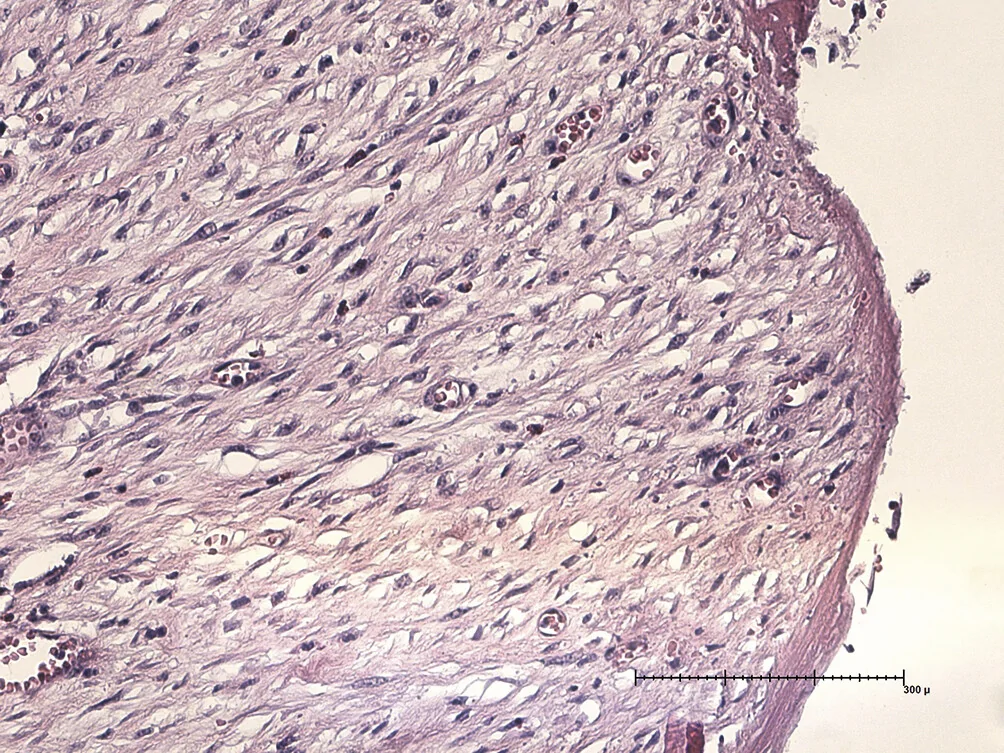
Inflammatory Fibroid Polyp. Loose mesenchymal tissue without glands, covered with fibrin.

**Fig. 8:**
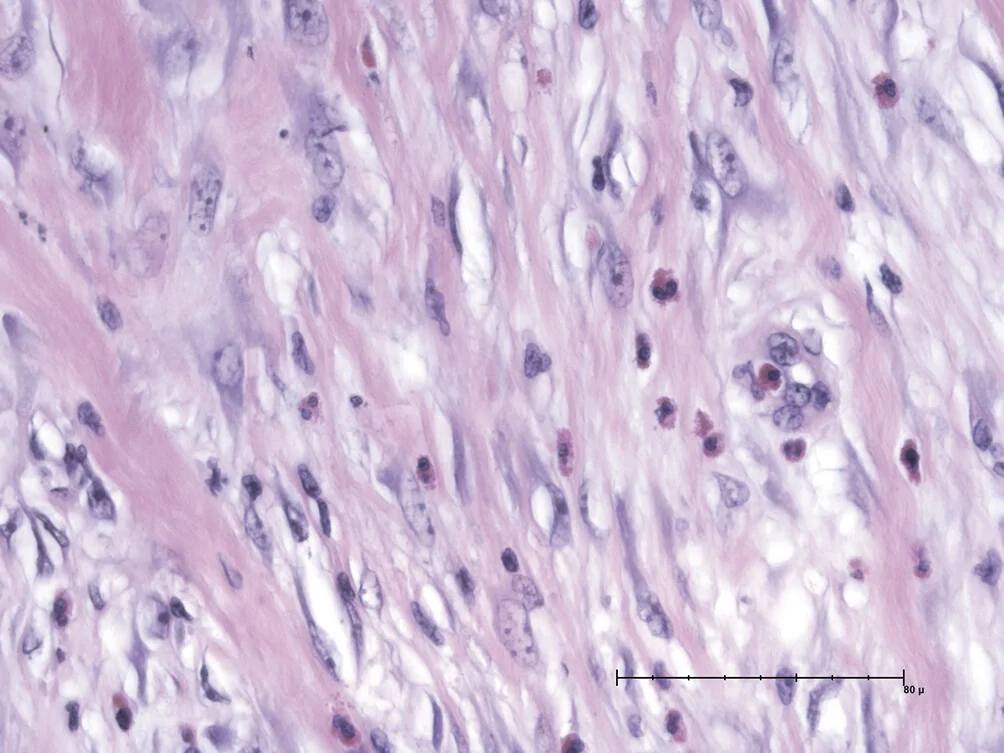
Inflammatory Fibroid Polyp. Loose chronic inflammatory cells, in particular eosinophils, in a proliferation of loose fibrovascular issue.

Due to the obstructing character of the big mass with symptomatic cholestasis and the risk of malignancy, which could not be ruled out with absolute certainty, the patient was referred to surgery. In the following she underwent left hemihepatectomy with lymphadenectomy and cholecystectomy. The final histopathological result revealed a 4.5 × 1.1 cm inflammatory fibroid polyp of the common and left hepatic bile duct with only small adherence to the duct wall. The further course of the patient was without complication, and she recovered rapidly. Almost all the parameters of cholestasis normalized after surgery. The serum bilirubin dropped from maximum 6.76 mg/dl to normal values ( Table 1 ). The follow-up after five years is uneventful. There is no regrowth of the polyp.

## Discussion

The presented case involves a 19-year-old female with recurrent upper abdominal pain, pruritus, jaundice, and laboratory/imaging evidence of cholestasis, with a mass involving the hepatic hilum and left hepatic bile duct. This presentation is highly atypical for IFPs, which predominantly affect middle-aged to elderly adults and rarely involve the biliary tract 1 2 3 11 . While extraintestinal IFPs have been reported, including a recent case in the renal pelvis confirmed by PDGFRA mutation analysis, biliary tract involvement remains exceedingly rare and is not well described in the literature 8 .

Clinically, IFPs may be asymptomatic or present with symptoms related to mass effect, such as pain, obstruction, or bleeding, depending on location and size 1 2 3 . In the gastrointestinal tract, lesions larger than 1 cm are more likely to harbor PDGFRA mutations and present with acute symptoms 11 . The current case's biliary involvement led to obstructive jaundice and cholestasis, mimicking malignant or other benign biliary tumors. Imaging modalities such as CT and MRI are sensitive for detecting mass lesions, but definitive diagnosis requires histopathological and molecular analysis 1 3 11 .

Management of IFPs is typically by endoscopic or surgical resection, which is curative, with no reported recurrence or malignant transformation 1 2 3 . In cases of biliary involvement, surgical excision may be necessary to relieve obstruction and obtain tissue for diagnosis. The benign nature of IFPs, even in unusual locations, underscores the importance of accurate diagnosis to avoid overtreatment.

This case expands the spectrum of IFPs to include possible biliary tract involvement in a young adult, emphasizing the need for awareness of atypical presentations and the utility of molecular diagnostics in confirming the diagnosis 7 8 9 11 .

In our case the SpyGlass DS single-operator cholangioscopy (SOC) proved to be a very useful method for macroscopic in situ evaluation of such a rare polyp. Nevertheless we were not able to remove the symptomatic IFP endoscopically. It is a very good tool for further evaluation of indeterminate biliary strictures, among other indications like therapy of difficult biliary stones. Direct visualization of the ductal system and obstructing lesions allows prediction of dignity. Turowski et al. could demonstrate for the SOC a sensitivity of 95.5 % and a specificity of 94.5 % in terms of predicting the dignity of a tumor 12 . In addition to visualization, it is also possible to take biopsies with SpyBite biopsy forceps under direct visual control. The SpyBite Max ameliorated the yield of biopsies due to twice as much tissue volume compared to the first generation SpyBite. The comparison between fluoroscopy-guided biopsies (standard forceps) and SOC-guided biopsies (SpyBite) revealed a better sensitivity and overall accuracy for the latter 13 . The initially taken brush cytology and fluoroscopy-guided biopsies in our case also did not lead to any histological result. Ultimately, the SOC-guided biopsies demonstrated the presence of a mesenchymal tumor. The inability to make a definitive diagnosis of an IFP is most likely due to its submucosal growth pattern.

A search in the PubMed database from 2004 up to 2025 for “inflammatory fibroid polyps” and “biliary” did not yield any relevant results. So we conclude that our case is the first reported IFP of the intrahepatic bile duct system. In the literature there is one case of an IFP in the  intrapancreatic bile duct just above the papilla of Vater, which also became evident through an obstruction of the bile duct and a cholestasis 14 .

In conclusion, this case report illustrates that stenosing biliary tumors can be observed even in very young patients and that IFP, a very rare benign mesenchymal tumor of the gastrointestinal tract, must also be considered. SpyGlass DS single-operator cholangioscopy can simplify diagnostic and therapeutic management.

Unfortunately, the endoscopic removal of the benign lesion remains challenging due to the restricted space in the bile duct. Nowadays, new surgical minimal invasive/robotic techniques allow a gentle surgical removal of the adjacent tissue.
